# The emergence of e‐cigarette retail shops in a regulated tobacco control environment

**DOI:** 10.1002/hpja.657

**Published:** 2022-09-19

**Authors:** Lucy Scott, Kahlia McCausland, Bruce Maycock, Jonine Jancey

**Affiliations:** ^1^ Collaboration for Evidence, Research and Impact in Population Health (CERIPH) School of Population Health, Curtin University Perth WA Australia; ^2^ College of Medicine and Health University of Exeter Exeter UK; ^3^ Enable Institute Faculty of Health Sciences, Curtin University Perth WA Australia

**Keywords:** healthy environments, healthy neighbouhoods, smoking

## Abstract

**Issue addressed:**

E‐cigarettes are of growing concern. We aimed to determine the location and characteristics of retail shops selling e‐cigarette products in Perth, Western Australia.

**Methods:**

Two phase study: (i) identifying all e‐cigarette retailers in the Greater Capital City Statistical Area of Perth; (ii) audit at the point‐of‐sale to assess products, promotions and shop characteristics (n = 41).

**Results:**

Ninety‐eight retailers selling e‐cigarette products were identified: 43 tobacconists (44%), 21 vape shops (21% – up from one shop in 2017), 14 supermarkets (14%), 12 service stations (12%) and 8 smoke shops (8%). The most common e‐cigarette product was non‐nicotine e‐liquid, available at 38 (93%) stores audited. Most stores sold parts of e‐cigarette devices (n = 25, 61%). Front counter displays were the most frequent form of promotion (n = 40, 98%). Vape shops differed from other retailers, having bar‐style layouts (n = 15, 71%), lounge areas (n = 7, 33%) and free e‐liquid samples (n = 17, 89%).

**Conclusion:**

The availability of e‐cigarette products from retail shops and particularly vape shops is increasing. E‐cigarette retailers are using traditional promotional techniques including point‐of‐sale displays to market their products, while vape shops are extending their appeal through bar style, lounge layouts and free trials.

**Implications for public health:**

Understanding the e‐cigarette retail store environment is essential for identifying emergent trends, potential regulations and future research.

**So what?:**

The e‐cigarette retail market in the Perth is growing, shops using traditional and new promotional techniques to market e‐cigarette products. Our findings identify a need for public health surveillance, regulations and legislation.

## 1 INTRODUCTION

Electronic cigarettes (e‐cigarettes) are battery‐operated, handheld devices that heat e‐liquid, also known as e‐juice, to produce an aerosol that the user inhales.^1^ E‐cigarettes, also known as vapes were commercialised in the mid‐2000s and have since evolved rapidly, resulting in a dynamic marketplace and a variety of e‐cigarette terminologies, designs and engineering.^1^

The two broad classes of e‐cigarettes are closed systems and open systems.^2^ Closed system either have a prefilled e‐liquid cartridge or ‘pod’ that attaches to a device and cannot be modified.^3^ In comparison, open system e‐cigarettes have cartridges that the user fills with e‐liquid.^3^ E‐liquid for open system devices is sold in bottles, with the consumer able to purchase any brand, or mix their own by adjusting flavouring additives and nicotine concentrations.^4^ Open systems include ‘build your own’ devices that have customisable parts.^4^

E‐cigarettes are marketed by the tobacco industry as a potentially ‘reduced risk’ alternative to traditional tobacco products,^5^, ^6^ even though their long‐term health and safety impact is not yet established.^1^ Being potentially less harmful than traditional tobacco products, which cause the premature death of up to two‐thirds of long‐term users,^7^ does not mean they are a safe product, and any use by non‐smokers will increase their risk of harm.^8^ There is also accumulating evidence that e‐cigarettes have adverse respiratory^9^ and cardiovascular health impacts.^10^

In Australia, young adults aged 18 to 24 are the group with the highest rates of ever use or experimentation with e‐cigarettes, with 63.9% of smokers and 19.6% of non‐smokers reporting having ever used an e‐cigarette.^11^ Between 2016 and 2019, the proportion of young adults who were current e‐cigarette users (including daily, weekly, monthly or less than monthly) almost doubled from 2.8% to 5.3%.^11^

E‐cigarette products are primarily sold through three channels: online, traditional tobacco retailers and vape shops. The majority of e‐cigarette users in Australia purchase their products online,^12^ however, brick and mortar vape shops are a large sector of the retail market in countries where e‐cigarettes are more freely available^13^ and an avenue for attracting new users, particularly youth.^14^ Vape shops provide a wide range of e‐cigarette brands, customisable devices and flavoured e‐juice, while offering an environment where consumers can discuss products and receive personalised advice.^15^, ^16^, ^17^ Products by the same manufacturers are often sold across channels, with online vendors selling in retail settings and vice versa.^18^, ^19^ In 2017, the Australian e‐cigarette industry was estimated to be worth $AUD50.1 million.^20^

While the relationship between traditional tobacco retailer density and sales promotion strategies are well documented,^21^, ^22^ there is a dearth of evidence on the e‐cigarette retail shop environment. Limited research on vapes retailers from New Zealand,^23^ the United States,^24^ United Kingdom^25^ and Canada^26^ has shown many vape shops use conventional promotion strategies, similar to those used for traditional tobacco products, including point of sale (POS) display and sales promotions. Research assessing the location of e‐cigarette shops and socio‐economic status provides mixed evidence, with New Zealand^23^ and United States^16^, ^27^ research suggesting that retail shops target those from lower‐income areas, while a US study in New York reported e‐cigarettes to be more readily available in economically advantaged communities.^28^

Australia provides a unique context with a strict regulatory environment for tobacco products and the prohibition of nicotine for use in e‐cigarettes.^29^ E‐cigarettes containing nicotine can only be obtained with an authorised prescription from a medical practitioner; it is an offence to manufacture, supply or sell nicotine without a licence or permit, as required by all states and territories.^29^ The regulation of non‐nicotine e‐cigarettes varies depending on state and territory legislation, with almost all jurisdictions having regulated the sale and promotion of non‐nicotine e‐cigarettes in a similar way to traditional tobacco products.^29^ As such, in most Australian states and territories it is illegal to sell e‐cigarettes to people under the age of 18 years, the use of e‐cigarettes is prohibited in existing smoke‐free areas, and there are restrictions on the advertising, marketing and display.^29^

Western Australia (WA) differs from other Australian states and territories in that the *Tobacco Products Control Act 2006* (WA) (s. 106) states a person ‘must not sell any food, toy or other product that is designed to resemble a tobacco product or packaging’.^30^ In 2014, the WA Supreme Court convicted a retailer of breaching the Act for selling e‐cigarettes that resembled a tobacco product.^31^ As a result of this case precedent, e‐cigarette devices cannot be sold in WA whether they contain nicotine or not. Since e‐cigarettes are not explicitly referred to in the *Tobacco Products Control Act 2006* (WA),^30^ the display and sale of the e‐liquid and e‐cigarette parts are not restricted in the same way as traditional tobacco products. E‐liquid is not considered a tobacco product because it does not contain tobacco, meaning there are no minimum age requirement laws for e‐liquid or e‐cigarette paraphernalia sold in WA.^30^

Despite strict regulations in WA, there are concerns e‐cigarettes have the potential to undermine current tobacco control efforts by attracting and supporting the initiation of nicotine addiction among youth and young adults.^3^ In addition, evidence indicates retailers are circumventing regulations by facilitating customers to purchase e‐cigarette devices.^32^, ^33^ The parts of open system e‐cigarettes (refillable devices, often consisting of customisable parts and accessories) do not always resemble a tobacco product when parts are sold separately and may not breach the legislation if one retail shop does not sell all parts to complete the device.^32^ E‐cigarette retailers are not required to have a licence; therefore, the extent to which e‐cigarettes are available and promoted through bricks and mortar shops in WA is unknown. This study is the first to assess the location, products, promotion, POS display and characteristics of shops selling e‐cigarette paraphernalia and e‐liquids in Perth, WA.

## 2 METHODS

### 2.1 Data collection

Data collection were conducted in two phases: (i) identifying all retailers that sell e‐cigarette products in the Greater Capital City Statistical Area (GCCSA) of Perth (ii) observational audit at the POS. This study was approved by the university Human Research Ethics Committee (HRE2019‐0351).

### 2.2 Mapping

The location of e‐cigarette retailers was compiled using a systematic internet search. The search engines included Google, Google Maps, Facebook and a combination of the search terms: ‘vape shop’, ‘vape store’, ‘e‐cigarettes’, ‘e‐cigarette retailer’, ‘Perth’. This search methodology has been identified as a valid method to identify vape shops, tobacconists and other retailers that sell e‐cigarette products.^34^, ^35^

The geographical area of the study was the GCCSA of Perth, as defined by the Australian Bureau of Statistics (ABS),^36^ represents the functional extent and urban area of WA's capital city.^36^ The 2020 Estimated Resident Population for the GCCSA was 2.125 million, approximately 79% of WA's total population.^37^

For inclusion in the study, retailers had to sell e‐cigarette products (open or closed e‐cigarette devices, e‐liquid, or accessories for open system devices such as tanks [part containing the e‐liquid], batteries and wire coils), have a store at a location with a public address and identify as a vape shop, smoke shop, tobacconist, supermarket or service station. The following definitions were applied:

1. Vape shop: a retailer that primarily or exclusively sells e‐cigarette products and accessories, and does not sell tobacco products (cigarettes, cigars, loose‐leaf tobacco).^38^
2. Smoke shop: a retailer that primarily sells smoking paraphernalia (eg water pipes, lighters, papers, grinders).
3. Tobacconist: a retailer that sells conventional tobacco products and accessories and includes newsagencies and convenience stores.

The socio‐economic advantage and disadvantage for each suburb was measured using the Index of Relative Socio‐Economic Advantage and Disadvantage (IRSAD) from the ABS Socio‐Economic Indexes for Areas.^39^, ^40^ The scores were reported in suburb‐based deciles, with 1 representing the most disadvantaged areas and 10 representing the most advantaged. The deciles were collapsed into three categories, low (decile 1–4), medium (decile 5–7) and high (decile 8–10).

### 2.3 Observational audit

Observational audits were conducted between July and September 2019, assessing products (e‐cigarette devices, e‐liquid, accessories and tobacco products) promotion (free samples, outside signage, quantity discounts, price discounts, loyalty programs), POS display and shop characteristics (layout, vaping in‐store, signage indicating minimum age to enter or purchase products). All vape shops were selected for inclusion (n = 21) and a random sample of other retailers: Tobacconist (n = 12); smoke shops (n = 4); supermarkets (n = 2) and service stations (n = 2) from low, middle and high IRSAD categories (n = 20) were included.

Observational data were collected through visual assessment of the shop by a single researcher (L.S.) and annotated using the Qualtrics app on a smartphone upon exiting the store. The observational survey was adapted from the Standardized Tobacco Assessment for Retail Settings survey^41^ and Vape Shop Standardised Tobacco Assessment of Retail Settings survey,^42^ with modifications reflecting the WA tobacco legislative environment. This instrument is available on request from the authors. The survey was piloted in three shops by a single researcher (L.S.) and reviewed by three researchers (J.J., B.M. and K.M.). The duration of retail observations was up to 10 min and visits occurred from Friday to Sunday.

The length of time the vape shops had been open was collected from retailers' Facebook page and websites. The researcher familiarised themselves with the shop's layout, enabling the researcher to enter the shop with an understanding of its layout, increasing efficiency in survey completion. The researcher used a smartphone to take a photo of the shop front and any outside promotional material. Evaluating retail shop environments using photography has been documented as a rapid method for capturing accurate information on marketing and advertisements.^43^

### 2.4 Data analysis

The location of e‐cigarette retailers was recorded in a spreadsheet and geocoded by the address using Google Maps. Descriptive analyses were conducted for the observational data using cross‐tabulations and frequencies to assess product type, promotion, POS display and shop characteristics by type of retailer. Survey data was exported from Qualtrics, and statistical analyses were conducted using SPSS v25.^44^

## 3 RESULTS/FINDINGS

### 3.1 Location

Google, Google Maps and Facebook search identified 96 retailers. Two additional tobacconist retailers selling e‐cigarette products were identified opportunistically while travelling to conduct the audits, making a total of 98 retailers: 43 tobacconists (44%), 21 vape shops (21%), 14 supermarkets (14%), 12 service stations (12%) and 8 smoke shops (8%). Of these, 39 (40%) retailers were classified as being in a low IRSAD, 28 (28%) in middle and 31 (32%) in high (Table 1).

**TABLE 1:** Type of e‐cigarette retailer located in the GCCSA of Perth by IRSAD (n = 98)

	Vape shop n (%)	Tobacconist n (%)	Smoke shop n (%)	Supermarket n (%)	Service station n (%)	Total n (%)
Low IRSAD	12 (57)	16 (37)	4 (50)	4 (29)	3 (25)	39 (40)
Middle IRSAD	4 (19)	14 (33)	0 (0)	7 (50)	3 (25)	28 (28)
High IRSAD	5 (24)	13 (30)	4 (50)	3 (21)	6 (50)	31 (32)
Total	21 (100)	43 (100)	8 (100)	14 (100)	12 (100)	98 (100)

The first vape shop was identified as opening December 2017, and at the time of the study (August 2019) 4 vape shops (19%) had been open for 2 years, 10 (48%) for 1 year (all opened in 2018) and 5 (24%) had been open for less than a year. Data were unable to be sourced for two vape shops.

### 3.2 Observational audit

#### 3.2.1 Shop characteristics

A noticeable characteristic of tobacconists, smoke shops, supermarkets and service stations that sold e‐cigarettes was the prominent display of the products, whereas vape shops had bar‐style counters (n = 15) lounge areas (n = 7) and vaping occurring in store (n = 9). Three (14%) vape shops had another vape shop directly adjacent, where one was the ‘main’ shop selling e‐liquid, accessories, and tanks, with the other exclusively selling the mods. One vape shop had different staff in both shops. The other two vape shops had the same staff serving customers between shops. These shops were identified as a separate shop in the mapping stage.

#### 3.2.2 Product

The most common e‐cigarette retail product was non‐nicotine e‐liquid, available in 38 (93%) of the observed shops. E‐cigarette device mods were sold at 23 (56%) shops and tanks were sold at 25 (61%) shops, while accessories (battery, coil cotton) were sold at 31 (71%) vape shops, four (100%) smoke shops, and three (25%) tobacconists. No retailers sold closed system e‐cigarette devices (such as Juul).

#### 3.2.3 Promotion

The promotion of products across the retailers included menu/poster of e‐cigarette flavours (n = 21, 51%), branded and/or promotional signage outside the shop (n = 24, 58%), quantity discounts and loyalty programs (n = 14, 34%). Only vape stores offered free e‐liquid samples (n = 17, 41%); had staff/customers vaping in store (n = 9, 22%) and offered zip/afterpay (n = 3, 7%). Signage indicating minimum age as 18 to enter or purchase products was present at 15 (71%) vape shops and 4 (100%) smoke shops. No tobacconist, supermarket or service station had signage indicating a minimum age for purchase (Table 2).

**TABLE 2:** Products, promotion and shop characteristics by retailer type (n = 41)

	Vape shop n = 21 n (%)	Tobacconist n = 12 n(%)	Smoke shop n = 4 n (%)	Supermarket n = 2 n (%)	Service station n = 2 n (%)	Total (n = 41) n (%)
Products						
E‐cigarette products						
E‐liquid	18 (86)	12 (100)	4 (100)	2 (100)	2 (100)	38 (93)
Mod (part containing battery)	19 (90)	3 (25)	1 (25)	0 (0)	0 (0)	23 (56)
Tanks (part containing e‐liquid)	19 (90)	3 (25)	3 (75)	0 (0)	0 (0)	25 (61)
Accessories (battery, coils, cotton)	19 (90)	8 (67)	4 (100)	0 (0)	0 (0)	31 (76)
Tobacco products						
Yes	0 (0)	12 (100)	0 (0)	2 (100)	2 (100)	16 (39)
Promotion						
Free e‐liquid samples	17 (81)	0 (0)	0 (0)	0 (0)	0 (0)	17 (41)
Menus/posters of e‐liquid flavours	15 (71)	2 (17)	0 (0)	2 (100)	2 (100)	21 (51)
Branded and/or promotional signage outside shop	21 (100)	3 (25)	0 (0)	0 (0)	0 (0)	24 (58)
A‐frame sign on street	8 (38)	1 (8)	1 (25)	0 (0)	0 (0)	10 (24)
E‐cigarette products visible from outside store	5 (24)	5 (42)	1 (25)	0 (0)	0 (0)	11 (27)
Quantity discounts	9 (43)	4 (33)	0 (0)	0 (0)	0 (0)	14 (34)
Sales discounts	9 (43)	0 (0)	0 (0)	0 (0)	0 (0)	9 (22)
Loyalty program	5 (24)	5 (42)	4 (100)	0 (0)	0 (0)	14 (34)
Zipay/Afterpay	3 (14)	0 (0)	0 (0)	0 (0)	0 (0)	3 (7)
Shop characteristics						
Bar‐style counter	15 (71)	0 (0)	0 (0)	0 (0)	0 (0)	15 (36)
Lounge area	7 (33)	0 (0)	0 (0)	0 (0)	0 (0)	7 (17)
Product display only (no seated areas)	6 (29)	12 (100)	4 (100)	2 (100)	2 (100)	26 (63)
Vaping in‐store (customers and/or staff)	9 (47)	0 (0)	0 (0)	0 (0)	0 (0)	9 (23)
Signage						
18+ to enter shop	7 (33)	0 (0)	4 (100)	0 (0)	0 (0)	11 (27)
18+ to purchase e‐cigarette products	9 (43)	0 (0)	4 (100)	0 (0)	0 (0)	13 (32)

#### 3.2.4 Product display – POS

All but one retailer displayed their e‐cigarette products, so they were visible from the counter (n = 40, 98%). Three‐quarters displayed e‐cigarettes at the checkout and behind the front counter (n = 29, 71%), and 8 (19%) retailers displayed products next to confectionary, while 16 (39%) had the e‐cigarette products in a locked/closed cabinet (Table 3).

**TABLE 3:** E‐cigarette products POS display, n = 41

	Vape shop n = 21 n (%)	Tobacconist n = 12 n (%)	Smoke shop n = 4 n (%)	Supermarket n = 2 n (%)	Service station n = 2 n (%)	Total n = 41 n (%)
Products visible to customers at front counter	21 (100)	11 (92)	4 (100)	2 (100)	2 (100)	40 (98)
At checkout/behind front counter	20 (95)	8 (67)	0 (0)	1 (50)	0 (0)	29 (71)
At checkout above front counter	3 (14)	1 (8)	0 (0)	1 (50)	2 (100)	7 (17)
Next to confectionary products	0 (0)	5 (42)	0 (0)	1 (50)	2 (100)	8 (19)
Next to smoking paraphernalia	1 (5)	3 (25)	4 (100)	1 (50)	0 (0)	9 (22)
In locked/closed cabinet	4 (19)	7 (58)	4 (100)	1 (50)	0 (0)	16 (39)

## 4 DISCUSSION AND INTERPRETATION

The e‐cigarette retail market in WA is nascent but growing rapidly, as seen by the exponential growth from 2017 to 2019, with e‐cigarette products available at vape shops, smoke shops, tobacconists, supermarkets and service stations across Perth. The mapping of e‐cigarette retailers showed a relatively even distribution across low, medium and high socio‐economic areas. However, over half of the vape shops (n = 12, 57%) were located in the lowest area of socio‐economic disadvantage. This is consistent with research showing that traditional tobacco retailers are more likely to be situated in lower socio‐economic areas^21^, ^22^ and a study in New Zealand that found tobacco outlets selling e‐cigarette products were typically located in areas with higher deprivation.^23^ However, it contrasts with US research, including a study in New York City that found e‐cigarettes were more accessible in higher income and predominantly white neighbourhoods.^28^ This indicates the need for research to identify associations between the socio‐economic location of e‐cigarette retailers, customer demographics and differentiation between stores, to identify any potential public health implications

This study identified strategies being adopted by e‐cigarette retailers to circumvent WA laws to facilitate e‐cigarette purchases. These strategies included having two separate shops adjacent to each other, one selling the mods (the main part of the device that contains the battery) and the other the tank (part of the device that holds the e‐liquid). Retailers creating two businesses to enable the purchasing of the whole e‐cigarette device through two transactions has been identified by previous WA research,^33^ suggesting a need for consideration of a more effective regulatory framework to close this loophole.

Prominent displays of e‐cigarette products were observed in almost every audited retail shop, with the concentration of displays and promotional materials located at the front counter, consistent with the findings from studies in New Zealand^23^ the United Kingdom,^25^ Canada^26^ and the United States.^24^ The promotion of e‐cigarettes in retail shops that can be visited by people under 18 years old (eg supermarkets, service stations and tobacconists) warrants increased attention, as these retailers provide an opportunity for product exposure to new youth markets. For example, some retailers displayed products next to confectionary items, which makes them appear innocuous, enjoyable and attractive, particularly to youth. Such displays and promotions have been banned for traditional cigarette products given the evidence linking exposure to tobacco POS promotions and youth smoking initiation.^45^

Almost half of the retailers (n = 19, 46%) had signage that indicated a minimum age of 18 to enter or purchase e‐cigarette products, however, currently there is no legislation to prohibit sales of e‐cigarette products to minors in WA. After decades of effective tobacco control measures in Australia, there are concerns that the promotion of e‐cigarettes may ‘renormalise’ tobacco use, initiate nicotine addiction among non‐smokers and youth, and perpetuate nicotine addiction rather than smoking cessation.^38^, ^39^ It is well established that tobacco companies have traditionally used confectionery and sweet flavours to entice young cigarette smokers^3^ and findings from an Australian study suggests that flavoured e‐cigarettes are appealing and preferred by young adults over non‐flavoured products.^46^

All retail outlets appeared to comply with the prohibition on the sale of nicotine e‐cigarettes. However, e‐liquids are not regularly tested for nicotine content to verify compliance. In New South Wales, Health Department inspectors seized more than 26 000 e‐cigarettes and e‐liquids either containing nicotine or labelled as such in January 2021.^47^ Furthermore, a WA study sampled 10 e‐liquids bought online and over the counter from Australian suppliers, finding nicotine present in 60%, despite being labelled as ‘non‐nicotine’ e‐liquid.^48^

Finally, vape shops were distinct from other retailers in the way they provided an inviting environment with bar‐style layouts, couches, free trials and opportunities to vape, essentially a community recreational space. These findings are consistent with other studies which reported vape shops offered an opportunity to socialise, reinforce a vaping identity,^49^ and sense of community,^32^ providing a wide range of e‐liquids and customisable devices, and discuss products and receive personalised advice.^50^

## 5 LIMITATIONS

Our study should be interpreted in the context of its limitations. First, we followed a previously validated online search method to identify vape shops for the mapping.^34^, ^35^ However, we did not identify all the e‐cigarette retailers (eg tobacconists, supermarkets and service stations) in the Greater Perth area, whose primary business is not e‐cigarette products. It still provides a snapshot of the activity in this area. Second, one observer collected all the audit data, however, subjectivity was reduced by using a standardised observational audit that had been reviewed by three researchers and pilot tested prior to data collection.

## 6 CONCLUSION

We found that the e‐cigarette brick and mortar retail market is expanding in the Perth GCCSA with vape shop numbers increasing from 1 in 2017 to 21 in 2019. Identified e‐cigarette retailers included vape shops, supermarkets, service stations, tobacconists and smoke shops. We found retail stores are using traditional tobacco promotional techniques to market their products. The specialist vape shops are creating casual attractive environments to encourage the trialling of e‐cigarette products as well as harnessing a sense of community. This growth in the e‐cigarettes retail environment represents a challenge for public health and policymakers as the number of retailers is increasing exponentially exposing these products to new users. Understanding this environment is essential for identifying emergent trends and providing evidence of the need for public health surveillance, potential regulations and legislation and future research.

## CONFLICT OF INTEREST

Jonine Jancey is an Editorial Board member of *Health Promotion Journal of Australia* and co‐author of this article. To minimize bias, they were excluded from all editorial decision‐making related to the acceptance of this article for publication. There are no other conflict of interest.

## Data Availability

The data that support the findings of this study are available on request.

## References

[1] National Academies of Sciences, Engineering, and Medicine . Public health consequences of e‐cigarettes. Washington, DC: The National Academies Press; 2018. Available from:. https://pubmed.ncbi.nlm.nih.gov/29894118/ 29894118

[2] Hsu G , Sun JY , Zhu S‐H . Evolution of electronic cigarette brands from 2013‐2014 to 2016‐2017: analysis of brand websites. J Med Internet Res. 2018;20(3):e80. 10.2196/jmir.8550 29530840 PMC5869180

[3] US Department of Health and Human Services . E‐cigarette use among youth and young adults. A report of the surgeon general. Altanta, GA: U.S. Department of Health and Human Services; 2016. Available from:. https://www.cdc.gov/tobacco/data_statistics/sgr/e-cigarettes/pdfs/2016_sgr_entire_report_508.pdf

[4] Levy T , Lindbom N , Sweanor T , Chaloupka F , O'Connor R , Shang C , et al. An economic analysis of the pre‐deeming US market for nicotine vaping products. Tob Regul Sci. 2019;5:169–81. 10.18001/TRS.5.2.8 32864395 PMC7454013

[5] British American Tobacco . Potentially reduced‐risk products. 2020. Available from: https://www.bat.com/ngp

[6] Philip Morris International . What are reduced‐risk products?. 2020. Available from: https://www.pmi.com/faq-section/faq/what-are-reduced-risk-products

[7] Banks E , Joshy G , Weber MF , Liu B , Grenfell R , Egger S , et al. Tobacco smoking and all‐cause mortality in a large Australian cohort study: findings from a mature epidemic with current low smoking prevalence. BMC Med. 2015;13(1):38. 10.1186/s12916-015-0281-z 25857449 PMC4339244

[8] Livingston CJ , Freeman RJ , Costales VC , Westhoff JL , Caplan LS , Sherin KM , et al. Electronic nicotine delivery systems or e‐cigarettes: American College of Preventive Medicine's practice statement. Am J Prev Med. 2019;56(1):167–78. 10.1016/j.amepre.2018.09.010 30573147

[9] Bircan E , Bezirhan U , Porter A , Fagan P , Orloff MS . Electronic cigarette use and its association with asthma, chronic obstructive pulmonary disease (COPD) and asthma‐COPD overlap syndrome among never cigarette smokers. Tob Induc Dis. 2021;19:23. 10.18332/tid/132833 33841062 PMC8025916

[10] Kavousi M , Pisinger C , Barthelemy J‐C , Smedt DD , Koskinas K , Marques‐Vidal P , et al. Electronic cigarettes and health with special focus on cardiovascular effects: position paper of the European Association of Preventive Cardiology (EAPC). Eur J Prev Cardiol. 2021;28:1552–66. 10.1177/2047487320941993 32726563

[11] Australian Institute of Health and Welfare [AIHW] . National drug strategy household survey 2019. Canberra: AIHW; 2020. Available from:. https://www.aihw.gov.au/reports/illicit-use-of-drugs/national-drug-strategy-household-survey-2019/contents/table-of-contents

[12] Australian Institute of Health and Welfare . Data tables: National drug strategy household survey 2019—2 Tobacco smoking supplementary tables. Table 2.34: Where people usually obtain their electronic cigarettes, by e‐cigarette user status, people aged 14 and over, 2016 to 2019 (col per cent). Canberra: AIHW; 2020. Available from: https://www.aihw.gov.au/reports/illicit-use-of-drugs/national-drug-strategy-household-survey-2019/data

[13] Braak DC , Cummings KM , Nahhas GJ , Heckman BW , Borland R , Fong GT , et al. Where do vapers buy their vaping supplies? Findings from the international tobacco control (ITC) 4 country smoking and vaping survey. Int J Environ Res Public Health. 2019;16(3):338. 10.3390/ijerph16030338 30691091 PMC6388194

[14] Berg CJ , Schleicher NC , Johnson TO , Barker DC , Getachew B , Weber A , et al. Vape shop identification, density and place characteristics in six metropolitan areas across the US. Prev Med Rep. 2020;19:101137. 10.1016/j.pmedr.2020.101137 32566458 PMC7298674

[15] Glantz SA , Bareham D . E‐cigarettes: use, effects on smoking, risks and policy implications. Ann Rev Public Health. 2018;39:215–35. 10.1146/annurev-publhealth-040617-013757 29323609 PMC6251310

[16] Dai H , Hao J , Catley D . Vape shop density and socio‐demographic disparities: a US census tract analysis. Nicotine Tob Res. 2017;19(11):1338–44. 10.1093/ntr/ntx063 28371830

[17] Yang JS , Lee E . A qualitative assessment of business perspectives and tactics of tobacco and vape shop retailers in three communities in Orange County, CA, 2015‐2016. Arch Public Health. 2018;76:57. 10.1186/s13690-018-0307-z 30349691 PMC6193290

[18] Levy T , Lindbom N , Sweanor T , Chaloupka F , O'Connor R , Shang C , et al. An economic analysis of the pre‐deeming US market for nicotine vaping products. Tob Regul Sci. 2019;5:169–81. 10.18001/TRS.5.2.8 32864395 PMC7454013

[19] Mackey TK , Miner A , Cuomo RE . Exploring the e‐cigarette e‐commerce marketplace: identifying internet e‐cigarette marketing characteristics and regulatory gaps. Drug Alcohol Depend. 2015;156:97–103. 10.1016/j.drugalcdep.2015.08.032 26431794

[20] Euromonitor International . Smokeless tobacco and vapour products in Australia. 2018. Available from: https://www.euromonitor.com/smokeless-tobacco-and-vapour-products-in-australia/report

[21] Wood L , Pereira G , Middleton N , Foster S . Socioeconomic area disparities in tobacco retail outlet density: a Western Australian analysis. Med J Aust. 2013;198(9):489–91. 10.5694/mja12.11539 23682892

[22] Melody SM , Martin‐Gall V , Harding B , Veitch MG . The retail availability of tobacco in Tasmania: evidence for a socio‐economic and geographical gradient. Med J Aust. 2018;208(5):205–8. 10.5694/mja17.00765 29540133

[23] Bateman J , Robertson LA , Marsh L , Cameron C , Hoek J . An analysis of e‐cigarette marketing in New Zealand tobacco retail outlets prior to legislative change. Nicotine Tob Res. 2020;22(7):1221–4. 10.1093/ntr/ntz226 31811294

[24] Garcia R , Allem JP , Baezconde‐Garbanati L , Unger JB , Sussman S . Employee and customer handling of nicotine‐containing e‐liquids in vape shops. Tob Prev Cessat. 2016;2(Suppl):7. 10.18332/tpc/67295 PMC5484151 28660255

[25] Eadie D , Stead M , Mackintosh AM , Macdonald L , Purves R , Pearce J , et al. E‐cigarette marketing in UK shops: an observational audit and retailers' views. BMJ Open. 2015;5:e008547. 10.1136/bmjopen-2015-008547 PMC4567676 26362665

[26] Hammond D , White CM , Czoli CD , Martin CL , Magennis P , Shiplo S . Retail availability and marketing of electronic cigarettes in Canada. Can J Public Health. 2015;106(6):e408–12. 10.17269/cjph.106.5105 26680433 PMC6972063

[27] Bostean G , Sanchez L , Lippert AM . Sociodemographic disparities in e‐cigarette retail environment: vape stores and census tract characteristics in Orange County, CA. Health Place. 2018;50:65–72. 10.1016/j.healthplace.2017.12.004 29414423

[28] Giovenco DP , Spillane TE , Merizier JM . Neighborhood differences in alternative tobacco product availability and advertising in New York City: implications for health disparities. Nicotine Tob Res. 2019;21(7):896–902. 10.1093/ntr/nty244 30452712 PMC6588385

[29] Greenhalgn E , Grace C , Scollo M . InDepth 18B: Electronic cigarettes (e‐cigarettes). In: Scollo MM , Winstanley MH , editors. Tobacco in Australia: facts and issues. Melbourne: Cancer Council Victoria; 2018. Available from:. https://www.tobaccoinaustralia.org.au/chapter-18-harm-reduction/indepth-18b-e-cigarettes/18b-9-legal-status

[30] Tobacco Products Control Act . Sect 106. 2006.

[31] Department of Health WA . Electronic cigarettes in Western Australia. 2016. Available from: https://ww2.health.wa.gov.au/Articles/A_E/Electronic-cigarettes-in-Western-Australia

[32] McCausland K , Jancey J , Leaver T , Wolf K , Freeman B , Maycock B . Motivations for use, identity and the vaper subculture: a qualitative study of the experiences of Western Australian vapers. BMC Public Health. 2020;20(1):1552. 10.1186/s12889-020-09651-z 33059652 PMC7559168

[33] McCausland K , Maycock B , Leaver T , Wolf K , Freeman B , Jancey J . “Is it banned? Is it illegal?”: navigating Western Australia's regulatory environment for e‐cigarettes. Int J Drug Policy. 2021;94:103177. 10.1016/j.drugpo.2021.103177 33713966

[34] Bostean G , Crespi CM , Vorapharuek P , McCarthy WJ . E‐cigarette specialty retailers: data to assess the association between retail environment and student e‐cigarette use. Data Brief. 2017;11:32–8. 10.1016/j.dib.2016.12.022 28127580 PMC5247279

[35] Kim AE , Loomis B , Rhodes B , Eggers ME , Liedtke C , Porter L . Identifying e‐cigarette vape shops: description of an online search methodology. Tob Control. 2016;25(e1):e19–23. 10.1136/tobaccocontrol-2015-052270 26205913

[36] Australian Bureau of Statistics . Capital City statistical area ‐ fact sheet. 2012. Available from: http://www.abs.gov.au/websitedbs/censushome.nsf/home/factsheetsgeography/$file/Greater

[37] idcommunity . Australia community profile Greater Perth. 2020. Available from: https://profile.id.com.au/australia/about?WebID=290

[38] Lee JGL , Orlan EN , Sewell KB , Ribisl KM . A new form of nicotine retailers: a systematic review of the sales and marketing practices of vape shops. Tob Control. 2018;27:e70–5. 10.1136/tobaccocontrol-2017-054015 29208738 PMC5988861

[39] Australian Bureau of Statistics . 2033.0.55.001 ‐ census of population and housing: socio‐economic indexes for areas (SEIFA), Australia, 2016. IRSAD. 2018. Available from: https://www.abs.gov.au/ausstats/abs@.nsf/Lookup/by%20Subject/2033.0.55.001~2016~Main%20Features~IRSAD~20

[40] Australian Bureau of Statistics . 2033.0.55.001 ‐ census of population and housing: socio‐economic indexes for areas (SEIFA), Australia, 2016. IRSAD interactive map. 2018. Available from: https://www.abs.gov.au/ausstats/abs@.nsf/Lookup/by%20Subject/2033.0.55.001~2016~Main%20Features~IRSAD%20Interactive%20Map~16

[41] Henriksen L , Ribisl KM , Rogers T , Moreland‐Russell S , Barker DM , Sarris Esquivel N , et al. Standardized tobacco assessment for retail settings (STARS): dissemination and implementation research. Tob Control. 2016;25(Suppl 1):i67–74. 10.1136/tobaccocontrol-2016-053076 27697950 PMC5099212

[42] Kong AY , Eaddy JL , Morrison SL , Asbury D , Lindell KM , Ribisl KM . Using the vape shop standardized tobacco assessment for retail settings (V‐STARS) to assess product availability, price promotions, and messaging in New Hampshire vape shop retailers. Tob Regul Sci. 2017;3(2):174–82. 10.18001/trs.3.2.5 29201950 PMC5708590

[43] Ilakkuvan V , Tacelosky M , Ivey KC , Pearson JL , Cantrell J , Vallone DM , et al. Cameras for public health surveillance: a methods protocol for crowdsourced annotation of point‐of‐sale photographs. JMIR Res Protoc. 2014;3(2):e22. 10.2196/resprot.3277 24717168 PMC4004156

[44] IBM Corp . Released 2017. IBM SPSS Statistics for Windows, Version 25.0. Armonk, NY: IBM Corp.

[45] Mantey DS , Pasch KE , Loukas A , Perry CL . Exposure to point‐of‐sale marketing of cigarettes and e‐cigarettes as predictors of smoking cessation behaviors. Nicotine Tob Res. 2019;21(2):212–9. 10.1093/ntr/ntx246 29126126 PMC6610161

[46] Jongenelis MI , Kameron C , Brennan E , Rudaizky D , Slevin T , Pettigrew S . E‐cigarette product preferences among Australian young adult e‐cigarette users. Aust NZ J Public Health. 2018;42(6):572–4. 10.1111/1753-6405.12842 30248716

[47] NSW Health . Illegal e‐cigarettes seized in crackdown. 2021. Available from: https://www.health.nsw.gov.au/news/Pages/20210218_01.aspx#:~:text=NSW%20Health%20is%20warning%20anyone,of%20illegal%20product%20last%20year

[48] Chivers E , Janka M , Franklin P , Mullins B , Larcombe A . Nicotine and other potentially harmful compounds in “nicotine‐free” e‐cigarette liquids in Australia. Med J Aust. 2019;210(3):127–8. 10.5694/mja2.12059 30636275

[49] Ward E , Cox S , Dawkins L , Jakes S , Holland R , Notley C . A qualitative exploration of the role of vape shop environments in supporting smoking abstinence. Int J Environ Res Public Health. 2018;15(2):297. 10.3390/ijerph15020297 29425117 PMC5858366

[50] Burbank AD , Thrul J , Ling PM . A pilot study of retail ‘vape shops’ in the San Francisco Bay area. Tob Prev Cessat. 2016;2(Suppl):6. 10.18332/tpc/65229 28393129 PMC5382868

